# Detection of Behavioral Anomalies in Medication Adherence Patterns Among Patients With Serious Mental Illness Engaged With a Digital Medicine System

**DOI:** 10.2196/21378

**Published:** 2020-09-10

**Authors:** Jonathan Knights, Zahra Heidary, Jeffrey M Cochran

**Affiliations:** 1 Otsuka Pharmaceutical Development & Commercialization Princeton, NJ United States

**Keywords:** digital medicine, mobile phone, entropy rate, Markov chains, medication adherence, contextual anomaly, psychiatric disorders

## Abstract

**Background:**

Adherence to medication is often represented in the form of a success percentage over a period of time. Although noticeable changes to aggregate adherence levels may be indicative of unstable medication behavior, a lack of noticeable changes in aggregate levels over time does not necessarily indicate stability. The ability to detect developing changes in medication-taking behavior under such conditions in real time would allow patients and care teams to make more timely and informed decisions.

**Objective:**

This study aims to develop a method capable of identifying shifts in behavioral (medication) patterns at the individual level and subsequently assess the presence of such shifts in retrospective clinical trial data from patients with serious mental illness.

**Methods:**

We defined the term *adherence volatility* as *“the degree to which medication ingestion behavior fits expected behavior based on historically observed data”* and defined a contextual anomaly system around this concept, leveraging the empirical entropy rate of a stochastic process as the basis for formulating anomaly detection. For the presented methodology, each patient’s evolving behavior is used to dynamically construct the expectation bounds for each future interval, eliminating the need to rely on model training or a static reference sequence.

**Results:**

Simulations demonstrated that the presented methodology identifies anomalous behavior patterns even when aggregate adherence levels remain constant and highlight the temporal dependence inherent in these anomalies. Although a given sequence of events may present as anomalous during one period, that sequence should subsequently contribute to future expectations and may not be considered anomalous at a later period—this feature was demonstrated in retrospective clinical trial data. In the same clinical trial data, anomalous behavioral shifts were identified at both high- and low-adherence levels and were spread across the whole treatment regimen, with 77.1% (81/105) of the population demonstrating at least one behavioral anomaly at some point in their treatment.

**Conclusions:**

Digital medicine systems offer new opportunities to inform treatment decisions and provide complementary information about medication adherence. This paper introduces the concept of *adherence volatility* and develops a new type of contextual anomaly detection, which does not require an a priori definition of *normal* and allows expectations to evolve with shifting behavior, removing the need to rely on training data or static reference sequences. Retrospective analysis from clinical trial data highlights that such an approach could provide new opportunities to meaningfully engage patients about potential shifts in their ingestion behavior; however, this framework is not intended to replace clinical judgment, rather to highlight elements of data that warrant attention. The evidence provided here identifies new areas for research and seems to justify additional explorations in this area.

## Introduction

**Figure 1:**
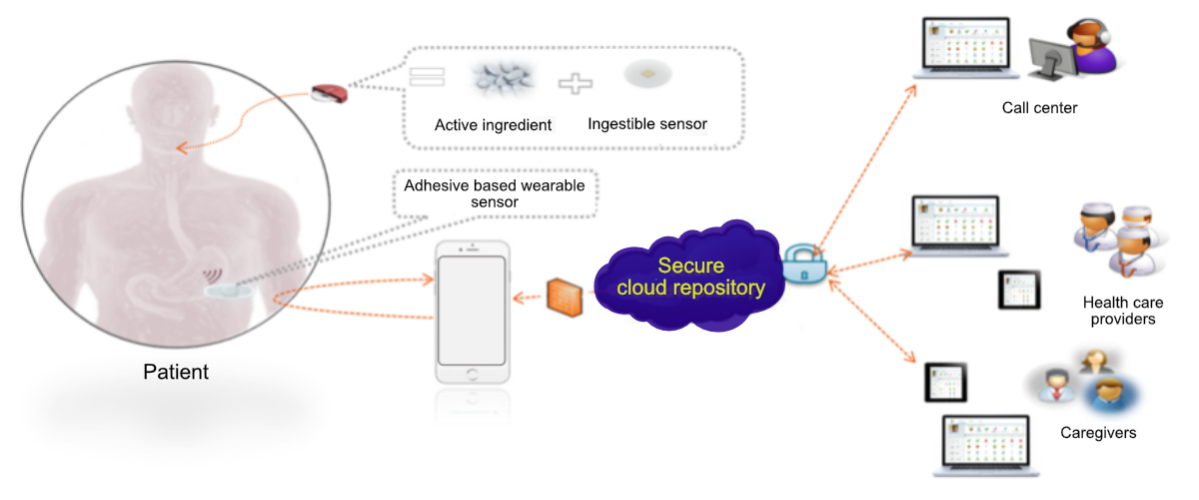
An overview of the digital medicine system (DMS). From left to right: Patient takes medication embedded with an ingestible sensor, which is activated in the stomach. The ingestible sensor is detected by a wearable sensor, which sends its collected information (including additional sensors not depicted) to the patient’s smartphone. The information is the passed on to a secure cloud infrastructure where it can be made available to appropriate members of the patient’s care team. Image reproduced under creative commons license from the article published in [18].

Lack of adherence to medication is a major issue, contributing to increased health care utilization and less favorable outcomes [1-3]. Common methods of measuring medication adherence such as the proportion of days covered or the medication possession ratio rely on claims data. Although useful for many applications, these methods do not provide objective evidence that the medication was ever taken. More objective measures such as electronic cap systems and electronic blister packs provide more granular observations at the event level but capture an interaction with the packaging, not the ingestion. Recently, the Food and Drug Administration approved the first ever digital medicine system (DMS) [4] to track medication ingestion in patients with serious mental illness (SMI). This system comprises an electronic sensor embedded in an active pharmaceutical, a wearable sensor (a patch), and a mobile application to collect and share data as appropriate (Figure 1). Systems such as the recently approved DMS hold promise for improving objective information available to patients, clinicians, and care teams, enabling better decision making. In this study, we sought to evolve the framework by which medication adherence information is leveraged in clinical decision making.

Adherence to medication is often represented in the form of a success percentage over a period of time, where success may refer to observations such as *prescription refills*, *bottle openings*, or, in the case of digital medicine, the detection of medication ingestions by the DMS. However, a single success rate over a period of time, regardless of the objectivity of the measure, may not—by itself—be sufficient to adequately assess a patient’s treatment adherence behavior. Although noticeable changes to aggregate adherence levels are certainly indicative of unstable medication-taking behavior, a lack of noticeable changes in these levels over time does not necessarily indicate stable, interpretable, medication adherence values. As demonstrated in the *Results* section, it is possible for anomalous shifts to be identified in day-to-day ingestion patterns in the absence of noticeable changes to the aggregate success rate. For instance, although a patient may be missing the same number of doses across defined intervals, one interval could have regularly interspersed misses whereas the other interval could have a single series of consecutive misses. Although the immediate clinical consequences of such differences would likely be dependent on compound properties such as half-life and therapeutic index, they could also indicate a more significant (potentially ongoing) behavioral change that is yet to manifest in an observable difference in the aggregate adherence value. Timely knowledge of such (potentially subtle) shifts in medication behavior may provide early opportunities for discussions and interventions.

One potential approach to detecting behavioral shifts in medication data is to apply contextual (or behavioral) anomaly detection. Anomaly detection, change point detection, or outlier detection refers to the task of identifying some part or pattern of data that is meaningfully different in some respects [5,6]. In general, there are 3 types of anomalies [5]: (1) point anomalies are individual points in the data that can be considered anomalous to the others, (2) contextual anomalies (also called conditional anomalies [7]) require the anomaly to be defined within a construct specific to the data, and (3) collective anomalies are extensions of point anomalies in that the presence of a set of points, which may not be individually anomalous, constitutes an anomaly when present together. Developing methodologies for anomaly detection typically requires specific consideration of the application at hand to frame the problem appropriately [5]. In the field of mental health, new and existing anomaly detection methods have begun to appear as an appealing option for a variety of applications, including relapse prediction [8-10], detection of illness [11,12], worsening cognitive impairment [13], motor skills [14], and anomalous traveling patterns [15,16]. For instance, by leveraging passively collected smartphone sensor data and digitally delivered patient surveys, Barnett et al [9] identified increases in the rate of anomalous behavioral patterns in 3 schizophrenia patients up to 7 days before a relapse. This study represents an important step in demonstrating the feasibility and applicability of individual-level anomaly detection for clinically relevant outcomes, albeit on a small sample size. Barnett et al [9] appropriately included in their discussion that the relapses “… quantified in the three subjects may not have reflected other potential trajectories and mechanisms that can lead to relapse,” supporting the value in developing other characterizations of behavioral anomalies from additional data sources. Using a natural language approach, Birnbaum et al [10] enrolled patients with recent onset psychosis and retrospectively combined social media data with medical records to identify anomalous linguistic patterns across monthly periods of relative health or relapse. Similar to Barnett et al [9], Birnbaum et al [10] note “Going forward, integrating multiple sources of digital data (sensors, social media, online searches) to predict mental health outcomes in clinical settings, could change the way clinicians diagnose and monitor patients …,” again speaking to the value of expanding the scope and depth of anomaly detection in the mental health space to inform links between behavioral changes and clinically meaningful outcomes at the individual level. Specific to the space of medication adherence, anomaly detection has been employed in patients with Parkinson disease, where observable changes in treatment pharmacodynamics (eg, gait patterns) were leveraged as a surrogate for medication compliance [17]. However, this approach cannot provide information on daily medication behavior, and when there are temporal lags between medication ingestion and effect (as is common in the mental health space), this type of approach may not provide an optimal alert window for detected anomalies. Although we are unaware of any similar studies validating specific behavioral anomalies to an intended clinical outcome, these initial studies demonstrate a growing ability to identify correlations in behavioral changes (anomalies)—at the patient level—to clinically meaningful observations, suggesting that further evidence and methods on novel data sources such as digital medicine will be of value to the clinical and research communities.

Previously published work has demonstrated that a first-order Markov model could describe digital medicine ingestion data at the population level [18]; however, this work did not address the temporal evolution of Markov chains at the individual level or provide a mechanism by which such information might be used proactively by clinicians, patients, or their support teams to aid in treatment decisions. This study addresses these 2 additional components. Furthermore, we defined the concept of a*dherence volatility* as *“the degree to which medication ingestion behavior fits expected behavior based on historically observed data.”*

Adherence volatility is formally represented as the longitudinal evolution of the entropy rate of a single (in this case binary) Markov chain generated from a patient’s medication ingestion data across treatment, where the success state indicates an observed ingestion on a given day (1) and an unobserved ingestion on a given day represents the unsuccessful state (0). The entropy rate has been utilized previously for characterizing behavioral data in rodents and can be thought of as “a quantification of the predictability of the next observation given the history of observations that occurred before it” [19]. When viewed longitudinally, the entropy rate metric can provide information as to shifts in both the marginal (stationary) and conditional dependence structures simultaneously, making it a promising measure by which to detect contextual (behavioral) anomalies.

On the basis of the aforementioned logic, an anomaly detection system was built that is computationally nonintensive and can be leveraged in real time to identify contextual behavioral anomalies, and shifts, at the individual level. Although we are aware of entropy rates previously explored for anomaly detection in complex dynamic systems [20,21], the current application differs in a critically distinct way: there is no *baseline truth* or external stimuli required in this system. A patient’s own evolving behavior (adherence volatility) is used to construct the expectation bounds for each future interval, eliminating the need for training or relying on a difference from a particular static reference sequence.

The results of this study provide some basic simulations to highlight behavioral anomalies and shift detection by leveraging the concept of adherence volatility. We also demonstrated the existence of such anomalies and behavioral shifts in previously collected clinical data. Although this is a retrospective analysis, we believe that knowledge of this approach, as well as its application and evolution, will be valuable to the medical and informatics communities as digital and objective medication adherence data become more prevalent in clinical practice. Finally, although the application of this approach is conceived based on medication ingestion data, there is no fundamental reason why the methods presented here could not be leveraged to implement the concept of *contextual anomaly detection* in any data that can be adequately represented as a stationary, irreducible Markov process.

## Methods

### Clinical Study Data

Ingestion data from two 8-week clinical trials (NCT02722967 and NCT02219009) of patients with SMIs (schizophrenia, major depression, and bipolar 1) being treated with a DMS was used. In this system (Figure 1), a patient-worn patch detects a signal from a digitized medication that contains an ingestible sensor. The patient-worn patch then transmits data to a mobile device and subsequently to a secure cloud infrastructure where it can be made available to clinical (and nonclinical) care teams to aid in decision making. We have previously provided descriptions of these studies [18] but recapitulated below for clarity.

Both of these studies provided smartphones with the appropriate DMS software preloaded and required male and female patients to be on stable, once-daily doses of oral aripiprazole. It was required that patients were deemed capable of using a DMS. During these studies, patients received only the digital versions of their stable oral aripiprazole dose. Both studies received human subject approvals from the Copernicus Group Institutional Review Board (One Triangle Drive, Suite 100, Research Triangle Park, North Carolina, United States), and subjects provided informed consent. Future use of clinical trial data for research was included in the consent for both studies, and no additional ethical approvals were required to leverage the data.

Study 1 was a multicenter, 8-week, open-label study with a primary objective of capturing the usability of the DMS by adult subjects with a diagnosis of schizophrenia with regard to their ability to independently (and successfully) replace their patch by the end of week 8 (NCT02219009). Patients were expected to perform 5 site visits following the screening period: baseline and weeks 1, 2, 3, and 8.

Study 2 was a multicenter, 8-week, open-label, single-arm, exploratory trial with the primary objective of assessing the functionality of an integrated call center for the DMS by adult subjects with primary diagnoses of schizophrenia, major depressive disorder, or bipolar 1 disorder (NCT02722967). This study consisted of 2 phases: a 2-week prospective phase and a 6-week observation phase. To progress to the 6-week observation phase, patients were required to have at least 50% patch data capture for the 7 days before the week 2 visit. Subjects who met this criterion were eligible to continue into the 6-week observation phase and would be expected to complete 4 total site visits (baseline and weeks 2, 4, and 8).

We leveraged data from patients (either study) who had more than 10 days of ingestion data (see the *Anomaly Detection from Adherence Volatility* section below: n=105/119) and defined Time on System as the difference between the minimum patch record or mobile application login and the maximum record from the same data sources. In some cases, the time on system may exceed the availability of patch and medication (eg, if last visit after 60 days and mobile application is still accessed in that timeframe), which will be annotated in the *Results* section where appropriate.

### Entropy Rate of a Markov Chain and Adherence Volatility

For a binary Markov chain (assumed to be stationary and irreducible), the entropy rate [19] is defined as


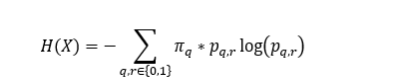


where *π_q_* is the stationary distribution of each state *q ∈{0,1}* representing 
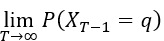
. The logarithm term in this implementation refers to the natural logarithm. For a subject *i* on day *T*, the observed data are represented as 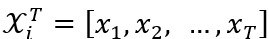, where *x_t_* ∈ {0,1} represents whether an ingestion was observed (1) or not (0) on day *t*. From the observed transition count data, *n_q,r_*, representing the counts of transitions q→r (q,r ∈{0,1}), the transition probabilities (TPs) are empirically estimated at any point in time using the maximum-likelihood definition of 
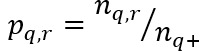
 [19]. The 2-state Markov chain for this subject, up to day *T*, is then represented by the transition matrix


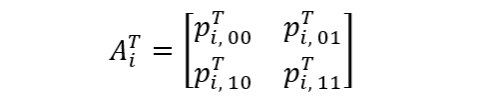


capturing the observed probabilities of ingestion successes and failures to be followed by success or failure. An estimate of the entropy rate of this Markov chain under these conditions is


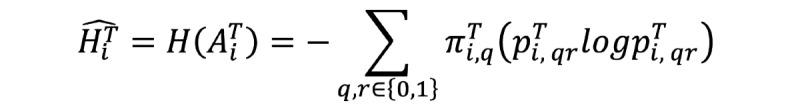


The stationary distribution 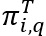 is estimated using the eigenvalue decomposition method on 
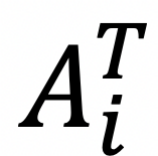
 [19,22]. Adherence volatility for subject *i* is represented as the longitudinal evolution of 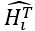.

The relatively short duration of data sets encountered in the current digital medicine application (generally around 60 days or less) makes it difficult to truly verify or explore the assumption of stationarity, and our assumption of irreducibility is based on the assertion that when applied to human behavior, no observed event eliminates the possibility of other events, provided that the behavior is observed over a sufficiently long timeframe. However, at short durations, it is possible for individual data sets to appear absorbing: for example, after 8 days, one patient’s data may be *11100000,* which has the illusion that the unsuccessful event may be absorbing. The accurate estimation of the entropy rate at this relatively short duration, even when the true order of the system is known (assumed first order in this case), has been shown to be challenging for multiple estimation methodologies [19]; however, the precision of estimation accuracy at any one point is not required to carry out contextual anomaly detection in this sense (discussed in the following section).

### Anomaly Detection From Adherence Volatility

It is acknowledged that individual alert systems could alternatively be constructed by directly tracking the occurrence rate of each particular sequence of interest in the data, for example, detecting an increasing number of consecutive missed doses. However, we propose to leverage the entropy rate for the evolving Markov chain as an ideal base candidate by which to identify anomalous behaviors in these data sets because it is a parsimonious metric that encompasses information on both the stationary and conditional distributions simultaneously.

The lack of ground truth (*normal*) makes leveraging existing anomaly detection systems challenging for application in this space. The expected behavior here should be allowed to shift over time to accommodate the actual shifting patterns in patient behavior, that is, anomalous behavior during one period should shift expectations for the future; no a priori assumptions are made about what *normal* behavior looks like, nor how many different shifts in behavior may occur over the (relatively short) observed sequences. These aspects of digital medicine ingestion data compromise the ability of existing techniques to identify anomalous shifts in the data from natural shifts.

**Figure 2:**
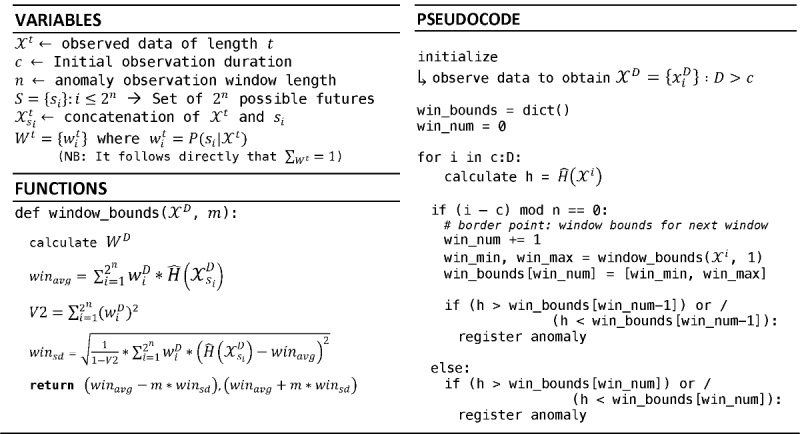
Contextual (behavioral) anomaly detection algorithm pseudocode.

Contextual anomaly detection in this study is built with an approach that could be classified as adaptive outlier detection. Figure 2 displays the pseudocode for the algorithm. Briefly, after an initial observation period (to allow for some TPs to be generated), the central tendency of the entropy rate observations for the next *n* days is calculated as a weighted average of all possible entropy rates *n* days into the future. For a binary Markov chain and an *n*-day observation window, there are 2*^n^* possible future states: The weights are calculated as the probability of each event given the historically observed data to that point. In this work, the expectation boundaries around the central tendency are set to 1 SD calculated from the observed weighted variance. Although there are existing methodologies and research around generating standard error bounds on estimates of entropy rate for a stochastic process [19], the intention here is to generate boundaries for the expected central tendency and variation in the empirical entropy rate over the next *n* days, simultaneously. After the expectation boundaries have been set for the observation window, the next *n* days are observed, logging an anomaly if the observed entropy rate goes outside the boundaries. At the end of each observation window, the boundary conditions are updated to include recent data; this process repeats until the treatment is completed. In this study, an initial period of 10 days was used, with a subsequent observation window of 5 days. These choices were made from practical and intuitive considerations so as to evenly divide a 1-month (30-day) treatment cycle.

To demonstrate proof of concept, this study highlights only one possible way in which the central tendency and boundary conditions can be set for the observation windows; however, the approach could be modified for specific use cases or as evidence suggests better alternatives. Further, we acknowledge that the full set of statistical properties of the weighted entropy rate distribution for each of the observation windows have not been described, but given that we define contextual anomaly detection here relative to observed data only, this formal characterization is not a prerequisite for application of the proposed approach.

From the definition outlined above (and in Figure 2), we define a *behavioral anomaly* as an observation window where the observed entropy rate goes outside the expectation range for any duration within that window and a *behavioral shift* as at least two consecutive anomalous windows. Although the aforementioned *behavioral anomalies* may provide potential indicators, the above definition may result in anomalous windows arising from a single missed ingestion; therefore, for the Results section, the majority of the focus will be on identifying *behavioral shifts*, as these are more robust indicators of change.

## Results

The salient results presented in this paper are observational and based on case studies. The intent is to highlight the presence and identification of behavioral anomalies and shifts, regardless of the level of aggregate adherence observed or the directionality of the anomaly with respect to ingestion success. Examples from simulations, as well as real clinical data, are provided and attempt to demonstrate the concepts with a broad range of observed adherence rates. Although these results have been generated retrospectively, the observations (anomalies and shifts) are reported in the moment for real-time access to clinical and care teams.

### Simulation Results

**Figure 3:**
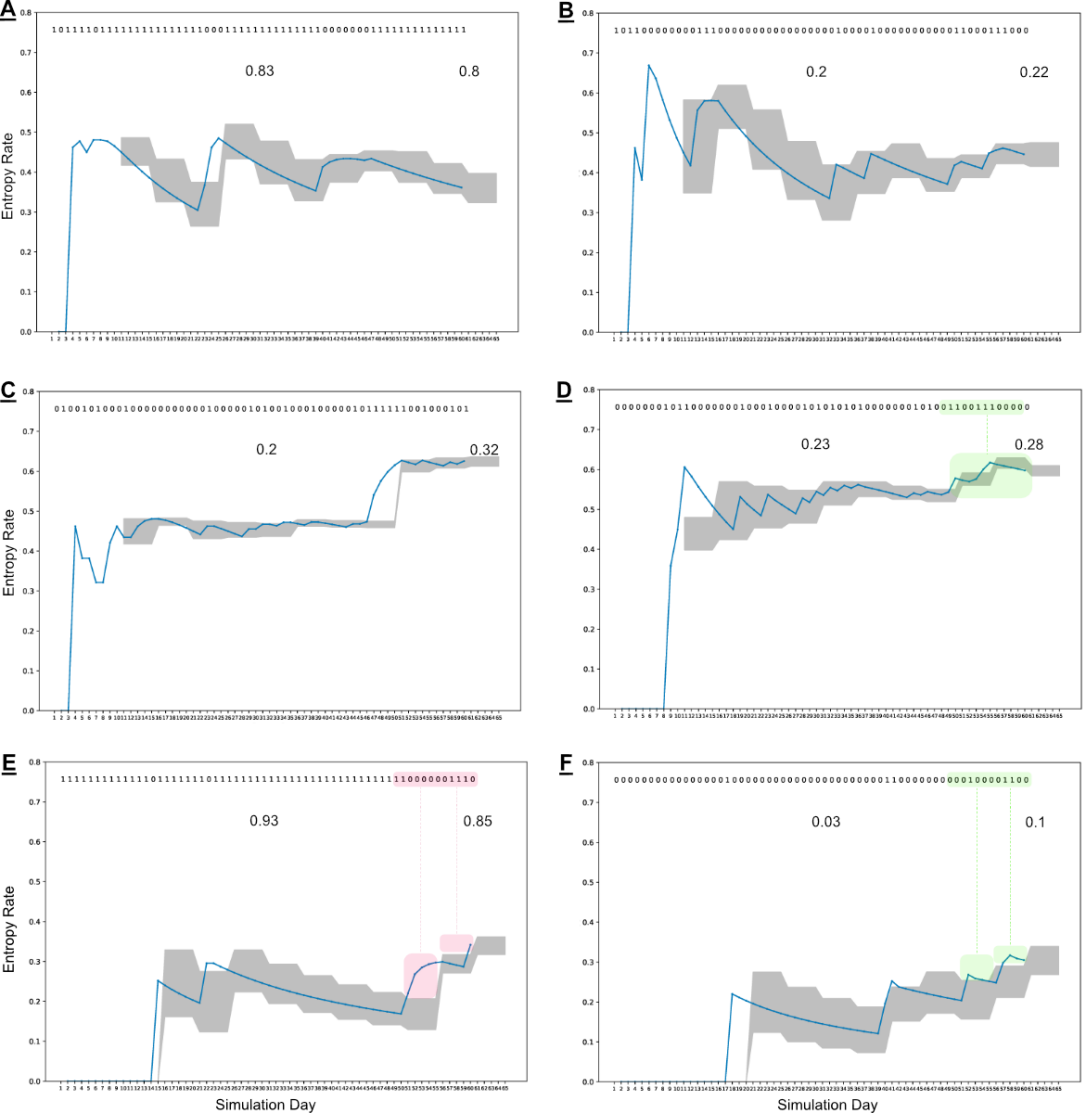
Adherence volatility plots for 6 simulated—representative—binary Markov chains over 60 days. The binary string at the top represents the underlying simulated data (0=unsuccessful, 1=successful). The 2 numeric values on the insets represent the observed aggregate adherence rates at 30 and 60 days, respectively. The blue line in the figures represents the calculated empirical entropy-rate based on the observed transition probability (TP) matrix to that day, whereas the shaded gray area represents the defined expected boundary across 5-day windows. Anomalies (single windows with deviations) are not highlighted in this figure; however, observed behavioral shifts (at least two consecutive windows with deviations) are identified in the data and trace plots by the green and red boxes. (A) Underlying TPs p_01_=.3, p_10_=.1 (expected success rate, ADHexp=0.75). The simulation has high observed adherence rates and 2 identified anomalies from observation windows 1 and 3. (B) Underlying TPs p_01_=.1, p_10_=.5 (ADHexp=0.17). The simulation has low aggregate adherence and only one anomaly identified from observation window 2. Both simulations (A) and (B) represent what could be considered as a stable observed ingestion behavior. (C) Underlying TPs p_01_=.3, p_10_=.5 (ADHexp=0.38). The simulation again has low observed aggregate ingestion success, but a dramatic anomaly at observation window 8, which seemingly restabilizes. (D) Again has underlying TPs p_01_=.3, p_10_=.5. Despite only 5% change in observed ingestion success rate, a behavioral shift is identified across observation windows 8-10 being driven by tighter groups of successes. Both (E) and (F) display behavioral shifts at the end of the simulations, with TPs p_01_=.5, p_10_=.1 (ADHexp=0.83) and p_01_=.1, p_10_=.3 (ADHexp=0.75) respectively. Groupings of unobserved events are driving the behavioral shift in (E), whereas groupings of successful events are driving the shifts in (F). In the last 2 examples, there is only a 5% to 7% change in observed success rates. These simulations are illustrative but provide insights that anomalies and shifts in this methodology are not required to represent shifts in the underlying system parameters, rather it detects contextual anomalies relative to what has been observed to date.

Figure 3 highlights 6 different simulated Markov chains, their resulting adherence volatility traces (blue lines), and their corresponding expected boundaries across 5-day observation windows (the gray-shaded region). Simulation durations of 60 days were chosen to mirror the available clinical data. For each of the simulations, the underlying TP matrix remains constant—the selected use cases represent only a few representative high- and low-adherence scenarios. This simplistic setup is presented for 2 primary reasons: (1) to demonstrate that even when the underlying behavior is not changing, there are noticeable shifts that can occur relative to historically observed data and (2) conversely, to highlight that an anomaly in this setting could arise for multiple reasons and does not inherently need to indicate a shift in the underlying system. For instance, simulations E and F both end treatment during an identified behavioral shift despite no change in the TPs. If these were observed in a clinical setting, additional data may be desirable before making a decision regarding the consistency of medication adherence. Simulations A and B, however, present stable ingestion patterns—demonstrated by the overlapping trace plots with the expectation boundaries over time (with the exception of an early anomaly)—despite very different levels of aggregate adherence.

All behavioral shifts (at least two consecutive anomalies) in the figures are identified by green or red boxes, with the corresponding sections of observed data highlighted. The green and red colors indicate if the driving factors for the detected shifts are anomalous patterns of dosing successes or unobserved doses, respectively. The behavioral shift in Figure 3 (D) is driven by increasing the frequency of dosing successes clumped together at shorter intervals than historically observed. In Figure 3 (E), the concentrated patches of zeros at the end are very irregular for that simulation to that point, and the presence of (at least) one more unobserved dose after only 3 consecutive observed doses is also irregular. These 2 instances in combination drive each of the last 2 windows to be tagged as anomalies, leading to its classification as a possible shift in behavior. Similar to simulation D, simulation F demonstrates successful events moving closer and appearing more frequently, albeit still at a very low rate.

### Clinical Data Results

**Figure 4:**
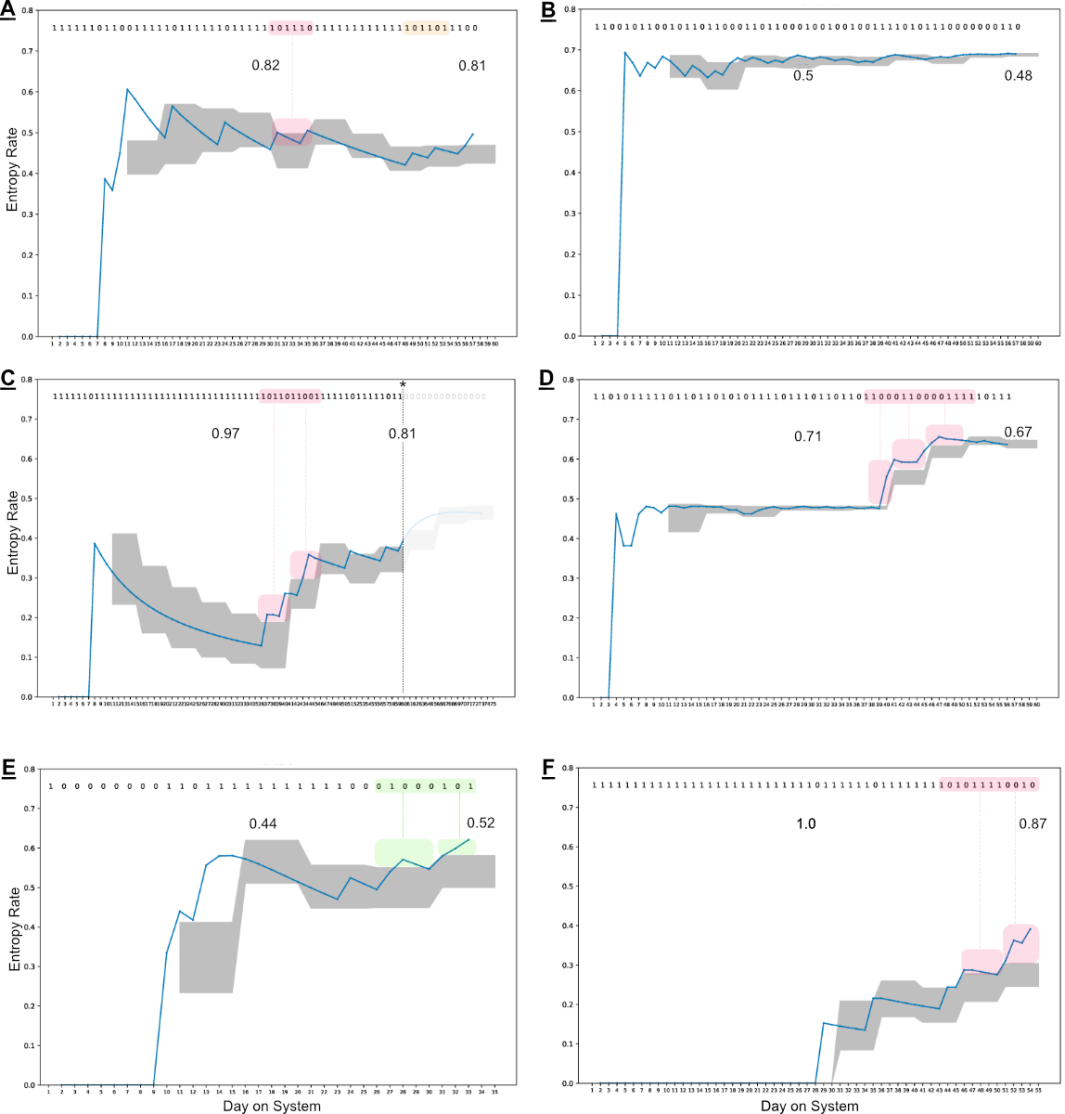
Clinical data from 6 subjects engaged with the DMS. The binary string at the top represents their observed ingestion data (0=unsuccessful, 1=successful). The 2 numeric values on the insets represent the observed aggregate adherence rates at their individual midway and end-of-treatment points. The blue line in the figures represents the calculated empirical entropy rate based on the observed transition probability matrix to that day, whereas the shaded gray area represents the defined expected boundary across 5-day observation windows. Anomalies (single windows with deviations) are not highlighted here; however, observed behavioral shifts (at least two consecutive windows with deviations) are identified in the data and trace plots by green or red boxes. (A) Demonstrates a behavioral shift driven by increasingly concentrated missed doses relative to historical observations. However, after this behavior is observed, the information is incorporated into future expectation, and when a similar pattern occurs again (light orange box over the observed data), it no longer results in an anomaly. (B) Has a low observed ingestion success rate but appears stable, with only one slight anomaly in window 2. (C) Demonstrates a behavioral shift starting in window 6 being driven by the presence of unobserved doses appearing more tightly clumped together, including consecutive unobserved doses appearing for the first time. Of note for Patient C, only the 60-day treatment window in which patches and medication were available is summarized in this analysis (identified up to the dashed line). (D) Demonstrates a behavioral shift across 3 windows, where sequences of 3 and 4 missed doses begin to surface. (E) Despite early difficulties to day 14, this patient appeared to be experiencing success in week 2 but ends treatment on a behavioral shift. (F) Ends treatment with 87% ingestion success; however, the number and frequency of missed doses in the last 30 days is still changing when the treatment ends. The adherence volatility data for (E) and (F) suggest that there are changes occurring in the patient’s ingestion behavior at the end of treatment that may warrant additional data collection despite their different success rates. Note: These events are generated purely on a statistical basis and would require clinical context and discussion to determine appropriate course of action (if any) when leveraged in real time. DMS: digital medicine system.

Figure 4 highlights selected patients across 2 clinical studies who were enrolled for 8 weeks of digital medicine treatment. These patients were selected to loosely recapitulate what was highlighted in the simulated data sets: the top 2 patients (A-B) had generally stable ingestion patterns at different ends of the adherence spectrum, and the middle 2 patients (C-D) end with stable behaviors but demonstrated behavioral shifts from the perspective of their day-to-day ingestion patterns: of note for Patient C, only the 60-day treatment window in which patches and medication were available is summarized in this analysis (identified up to the dashed line). Finally, the last 2 patients (E-F) demonstrated ongoing behavioral shifts with respect to their historical data, without much change in the aggregate adherence values. All behavioral shifts are identified in the plots as red or green boxes along with the corresponding subsequences in the observed data. Patient A demonstrates a behavioral shift driven by increasingly concentrated missed doses relative to historical observations. However, after this behavior is observed, the information is incorporated into future bounds, and when a similar pattern occurs again (light orange box over the observed data), it no longer results in an anomaly. Patient C demonstrates a behavioral shift starting around day 37, which is driven by the presence of unobserved doses appearing more tightly clumped together, including consecutive unobserved doses appearing for the first time. Patient D demonstrates a strong behavioral shift across 3 observation windows (15 days): in this scenario, sequences of 3 and 4 missed doses begin to surface. Of particular interest in the clinical data is Patient F. Despite successfully registering 87% (47/54) of prescribed ingestions across the treatment period, when viewed from the perspective of the adherence volatility plots, the last 30 days of treatment suggest a continuing shift in ingestion behavior that has not stabilized by the time treatment is over. It is unclear whether the ingestion patterns would have continued to shift in this direction, but this is a very clear example from the available clinical data of where this approach can add a unique perspective to clinical intuition: a medication adherence rate of 87% is considered high, but the adherence volatility data suggest that there are significant changes occurring in the patient’s observed ingestion behavior.

Table 1 shows the count distribution of observed behavioral shifts in each patient included in the analysis: 22.8% (24/105) of patients had no behavioral shifts observed during treatment, 71.4% (75/105) of patients had only 1 behavioral shift observed, and 5.7% (6/105) of patients had 2 behavioral shifts observed across their treatment (NB: these counts include total *Days on System*, not just the treatment window as is discussed in Figure 4). This table is included to provide information on the current scale of identified behavioral shifts across patient data; however, given the relatively small sample size for this analysis and the current inability to provide context around these behavioral shifts, no comparisons are made across demographics.

**Table 1:** Count distribution of observed behavioral shifts in clinical data.

Number of shifts	Participants, N	Fraction pop
0	24	0.23
1	75	0.71
2	6	0.06

## Discussion

Medication adherence is an important issue in chronic conditions. Although tools for monitoring adherence to medication have seen dramatic improvements—including the first approved DMS for patients with SMI—there have not been parallel advances in data products and algorithms to accompany them. In this study, we present the concept of *adherence volatility* and provide a complementary anomaly detection system that focuses on contextual *behavioral anomalies*. Anomaly detection in this framework does not require an a priori definition of what normal means and allows expectations to evolve with shifting behavior such that observing an anomaly in one observation period informs the expectations of subsequent observation periods. Further, this framework is not intended to replace clinical judgment: anomalous data patterns detected here are intended to highlight elements of data that may warrant attention from the patient’s clinical and support teams who would determine the best course of action (if any) when identified in real time.

The DMS leveraged in this study requires compliance with both a wearable and an ingestible component to generate a successful signal. Thus, although a successfully observed signal is a robust, objective indicator of ingestion, an unobserved ingestion may arise from multiple scenarios. Although this makes the exact interpretation of anomalies difficult here, the current iteration represents when ingestion behavior, at the system level, is momentarily different or shifting, which we believe is a beneficial starting point given that future iterations could become more specific as to which component is driving a detected anomaly, or collect patient feedback in the moment if an anomaly is detected.

We leveraged the entropy rate of a Markov chain as the basis to build the proposed anomaly detection system. Despite its *catch all* nature in terms of what types of anomalous patterns can be detected, observing the evolution of this metric over short durations will undoubtedly contain both expected and anomalous shifts in observed values: deciphering the expected from the anomalous shifts in this metric, and at these scales, is not a task for which current standard anomaly detection systems are equipped to succeed. We also frame the anomaly detection problem as a contextual problem, which is not dependent on accurate point estimates of the entropy rate. Rather, the approach is concerned with the magnitude of relative changes at a given point. This change alleviates the downstream complexity of generating accurate point estimates of the entropy rate for short durations [19].

From an interpretation standpoint, a detected anomaly or shift does not directly indicate either a true shift in the underlying system or a change in aggregate adherence levels per se. However, this indicates that the current observed patterns are not consistent with expectations based on data generated up to that point. The simulated sequences presented in this paper (Figure 3) highlighted this by demonstrating anomalies and shifts from evolving Markov chains with stable underlying transition matrices. The choice to adopt a weighted average and variance approach was used to highlight the initial system and demonstrate the proof of concept; however, this framework and approach may evolve over time as additional data and evidence become available to support other measures, or boundary conditions, which may be more effective at detecting certain types of clinical scenarios. The same would also apply for the choice of observation window duration: as clinical outcomes and observations are collected in conjunction with digital medicine data, optimal observation window durations may arise beyond the currently displayed 5-day duration.

Data available for this study were from once-daily dosing of a single medication (aripiprazole) for patients who were already on stable doses. The homogeneity of the patient population from a stability and dosing regimen standpoint and a lack of in-the-moment feedback or clinical exploration into observed behavioral shifts are clear limitations to the generalizability of this study. Formal exploration of how these methods and concepts interact with more complicated underlying dynamics and clinical outcomes are additional opportunities for future research. Despite the current inability to provide insight into the clinical relevance of detected anomalies and shifts in the presented data, we believe this study demonstrates that focusing solely on aggregate adherence levels misses opportunities to effectively interact with patients and make the most informed treatment decisions. It was particularly interesting to find examples of both stable and shifting adherence volatility behaviors at high- and low-adherence levels despite the lack of heterogeneity in the available data.

DMSs, such as the one leveraged in this study, offer new opportunities to inform treatment decisions and provide complementary information about medication adherence. The anomaly detection framework that has been developed identifies one way of leveraging such information to improve patient care by identifying potentially meaningful changes in medication behavior over time. This unique approach to medication behavior also opens the door to new areas of potential research. Although much work remains to be carried out to determine (and validate) how and when to leverage such information to inform clinical care, the evidence provided here, along with the growing body of evidence supporting the potential for applying anomaly detection to advance the goal of personalized care, seems to justify continued explorations.

## Abbreviations

DMS: digital medicine system
SMI: serious mental illness
TP: transition probability
